# Adaptive Expertise of College English Teachers in the Era of Artificial Intelligence: A Grounded Theory Approach

**DOI:** 10.3390/bs16040476

**Published:** 2026-03-24

**Authors:** Qi Zhou, Luming Hu, Xintong Zou, Xuemin Zhang

**Affiliations:** 1 College of Education for the Future, Beijing Normal University, Zhuhai 519087, China; 202132120096@mail.bnu.edu.cn; 2 Center for Foundational Courses, Beijing Institute of Technology, Zhuhai 519088, China; 3 Department of Psychology, School of Arts and Sciences, Beijing Normal University at Zhuhai, Zhuhai 519085, China; 4 Bay Area School of Applied Psychological Sciences, Beijing Normal University at Zhuhai, Zhuhai 519087, China; 5 Beijing Key Lab of Applied Experimental Psychology, Faculty of Psychology, Beijing Normal University, Beijing 100875, China; 6 State Key Laboratory of Cognitive Neuroscience and Learning & IDG/McGovern Institute for Brain Research, Beijing Normal University, Beijing 100875, China

**Keywords:** artificial intelligence, adaptive expertise, college English teachers, qualitative analysis

## Abstract

The rapid advancement of artificial intelligence (AI) and the continuous reform of English education in China have greatly reshaped the professional requirements for college English teachers. In contrast to the predominance of general teacher perspectives in adaptive expertise research, college English teaching offers a discipline-specific context in which adaptive expertise becomes particularly salient in response to AI-related pedagogical change. This study adopts a grounded theory approach to explore the qualities that college English teachers should possess in the era of artificial intelligence. A five-dimensional dynamic model of adaptive expertise was developed, consisting of knowledge expertise, competence expertise, vision expertise, emotional expertise, and development expertise. It shows that knowledge expertise embodies the integration of multiple knowledge domains required in the AI era; competence expertise emphasizes the ability to design and implement AI-enhanced instruction; vision expertise reflects global and future awareness in AI-technology integration; emotional expertise sustains resilience and motivation amid technological change; and development expertise promotes lifelong learning and innovation. These dimensions transfer, enhance, and inspire one another, forming a closed and self-reinforcing loop. It enriches the understanding of teacher professionalism in the AI era and offers a framework for cultivating AI-resilient expertise in higher education.

## 1. Introduction

### 1.1. College English Teaching in the AI Era

The rapid emergence of artificial intelligence (AI) technologies has profoundly reshaped the landscape of higher education. In China, college English education, once served as a cornerstone of linguistic and cultural learning, is now facing new opportunities and challenges brought about by AI integration. Under this rapidly changing context, college English teachers are expected to possess not only linguistic and pedagogical expertise but also technological literacy, intercultural awareness, and reflective capacity to adapt to AI-enhanced teaching environments. In other words, teachers must develop “adaptive expertise”—the capacity to flexibly apply knowledge, respond creatively to novel situations, and continuously reconstruct their professional practices in dynamic contexts (Bransford et al., 2000). Adaptive expertise enables teachers to balance efficiency in routine tasks with innovation in unfamiliar scenarios, a skill increasingly vital in the age of AI when instructional content, student needs, and learning tools evolve rapidly.

Despite growing discussions on teacher professionalism and digital competence, few empirical studies have examined what adaptive expertise means specifically for college English teachers in the AI era. Most existing studies on adaptive expertise have focused on medicine, engineering, or general teacher education (Carbonell et al., 2014), which often adopt a general teacher perspective that pays insufficient attention to discipline-specific pedagogical contexts, while research in applied linguistics rarely addresses how language teachers develop and exercise adaptive expertise when confronted with intelligent technologies. Recent studies in language education have explored teachers’ professional agency, emotional resilience, and reflective practice (Farrell, 2018; Lee & Yuan, 2021), yet the dynamic integration of these traits with AI-related competencies remains underexplored. Moreover, existing frameworks for evaluating English teachers—such as the TESOL and ACTFL standards—emphasize linguistic proficiency and pedagogical competence but pay limited attention to teachers’ adaptability to emerging technologies and sociocultural change.

From a discipline-specific perspective, college English teaching provides a particularly informative context for examining adaptive expertise in the era of artificial intelligence. Language teaching, which primarily focuses on interaction and meaning-making, has increasingly been reshaped by recent advances in AI technologies. Moreover, in the context of Chinese higher education, ongoing curricular reform and the rapid incorporation of digital and intelligent tools have further increased the need for college English teachers to flexibly adjust their pedagogical methods and instructional decisions. These features support the selection of college English teachers as an appropriate research group for exploring how adaptive expertise is constructed and sustained in AI-enhanced educational settings.

### 1.2. The Current Study

The present study seeks to bridge the gap between the theoretical understanding of adaptive expertise and the practical realities of English teaching in AI-driven environments. It argues that adaptive expertise among college English teachers should not be viewed as a static set of skills but as a dynamic system of interconnected competencies shaped by technological, emotional, and cognitive factors. Understanding these components is essential for redefining teacher education, professional development, and curriculum reform in China’s AI-empowered higher education landscape. Therefore, this study aims to identify the key components of adaptive expertise among college English teachers in the age of artificial intelligence and to construct a theoretical model that explains how these components interact to support teacher adaptability and professional growth. The following research questions guide the inquiry:

(1) What are the core components of adaptive expertise among college English teachers in AI-mediated educational contexts?
(2) How do these components interact to form a dynamic model of adaptive expertise that reflects the professional demands of the AI era?

## 2. Literature Review

### 2.1. Adaptive Expertise

The term “adaptive expertise” was first proposed by Japanese scholars Hatano and Inagaki. It refers to the ability of practitioners to not only efficiently complete routine tasks (routine expertise) but also to flexibly deal with non-structured and dynamically changing professional challenges (Hatano & Inagaki, 1986). Bransford et al. were among the first to recognize the enlightening significance of “adaptive expertise” for educational and learning research. In contrast to routine expertise, which prioritizes efficiency through repetition, adaptive expertise embodies the capacity to transfer knowledge, reframe problems, and generate innovative solutions in dynamic contexts (Bransford et al., 2000). Lin et al. (2005) argued that adaptive expertise evolves through reflective engagement with diverse problems, rather than the accumulation of knowledge alone. Studies have further identified several components of adaptive expertise: deep conceptual understanding, cognitive and metacognitive flexibility, emotional resilience, and motivation for continual learning (Carbonell et al., 2014; Mercier & Higgins, 2013).

In the AI era, adaptive expertise has regained its importance. Teachers are required to keep up with technological and pedagogical innovations while maintaining humanistic values. Thus, adaptive expertise is a dynamic capability that integrates cognitive, emotional, and technical dimensions to support effective teaching amid educational transformation.

### 2.2. Adaptive Expertise Among Language Teachers

While adaptive expertise has been studied extensively in general teacher education, research focusing on language teachers remains limited. Language teaching inherently requires integrating linguistic, cultural, and pedagogical dimensions. Teachers must continuously adapt to students’ linguistic diversity, evolving curricula, and shifting cultural norms, which closely correspond to the concept of adaptive expertise. Byram (1997) proposed that effective language teaching requires intercultural sensitivity and empathy, both of which are central to adaptive performance. In applied linguistics, related terms such as teacher cognition (Borg, 2003), agency (Priestley et al., 2015), and reflective practice (Farrell, 2018) share the conceptual foundation with adaptive expertise. These studies emphasize that language teachers’ professional competence develops through reflective interaction with classroom situations and sociocultural contexts.

However, the rise of artificial intelligence (AI) has accelerated the transformation of teacher roles and competencies across disciplines. Intelligent tutoring systems, automated writing feedback, and generative AI applications have redefined classroom interactions and instructional design (Zawacki-Richter et al., 2019). Teachers are now expected to move beyond content delivery and act as facilitators of ethical, critical, and human-centered learning (Holmes et al., 2019). AI technologies improve students’ learning efficiency. But at the same time, they may also risk diminishing their linguistic engagement and critical thinking. Consequently, teachers must acquire not only technical proficiency but also adaptive capacities to design meaningful, humanistic, and culturally grounded learning experiences. The integration of technology, pedagogy, and teacher adaptability has become an urgent need for current educational research.

### 2.3. Dynamic Teacher Expertise in the AI Era

Professional frameworks such as Teachers of English to Speakers of Other Languages (TESOL, 2018) and the American Council on the Teaching of Foreign Languages (ACTFL, 2012) provide established benchmarks for defining English teachers’ competencies in linguistic proficiency, pedagogy, and intercultural communication. However, these standards largely reflect a pre-AI paradigm that conceptualizes teachers’ competence as stable and measurable. Adaptive expertise highlights the need for flexibility, continual learning, and contextual responsiveness. In the Chinese context, the College English Guidelines formulated by the Ministry of Education (2020) emphasize cultivating critical thinking, intercultural competence, and digital literacy, echoing the need for adaptability in rapidly changing learning environments.

Integrating international and domestic standards, this study suggests that teacher expertise is fluid and multidimensional, combining linguistic, pedagogical, emotional, and technological adaptation. However, little empirical work has examined how Chinese college English teachers cultivate such competence in AI-driven classrooms. This study seeks to fill that gap by identifying the core dimensions of adaptive expertise and constructing a context-sensitive framework to explain how college English teachers sustain growth and adaptability in technology-mediated learning environments.

## 3. Methodology

### 3.1. Participants

The study involved 40 college English teachers recruited from ten universities across different regions of China. A purposeful sampling strategy with maximum variation (Cresswell, 2013) was adopted to ensure diversity in terms of institutional type, professional title, educational background, and teaching experience. Informed consent was obtained prior to the interviews. Ethical approval for this research was granted by the authors’ affiliated institution. Data were collected through semi-structured interviews, conducted either in person or online, depending on the participants’ schedules and locations. The demographic characteristics of the 40 college English teachers are summarized in Table S1 in the Supplementary Materials. Each interview lasted for 25–30 min. All interviews were conducted in Chinese and recorded with the participants’ consent. The recordings were transcribed word-for-word, and the Chinese transcripts were subsequently translated into English by the first author. To ensure accuracy and linguistic equivalence, the translations were checked by a bilingual colleague specializing in applied linguistics.

### 3.2. Interview Questions

The interview questions were developed based on a review of relevant literature on adaptive expertise, teacher professional development, and technology integration, combined with the researchers’ prior experience in college English teaching, with the aim of exploring how teachers understand and experience adaptive expertise. Instead of relying on a fixed interview schedule, the interview emphasized openness and flexibility, allowing participants to express their own understandings of professional change and teaching adaptation. This flexible and open-ended interview design is consistent with qualitative and grounded theory research, which emphasizes participants’ meanings and inductive category development (Corbin & Strauss, 1990; Charmaz & Thornberg, 2021). Specifically, the interview outline was designed around three domains: teachers’ perceptions of AI-related change, their adaptive teaching practices, and their professional learning and emotional responses to technological transformation. During the data collection process, the interview questions were used as guiding prompts rather than rigid scripts. Follow-up and probing questions were employed flexibly in response to participants’ answers to encourage deeper exploration of emerging issues. The detailed interview questions are provided in Section S2 of the Supplementary Materials.

### 3.3. Data Analysis

To examine the adaptive expertise that college English teachers should possess in the AI era without an existing framework, this study employed a grounded theory approach (Corbin & Strauss, 1990). The method was chosen because teacher competence in AI-impacted education remains a developing area with limited empirical modeling. Grounded theory allowed theoretical patterns to emerge inductively from teachers’ experiences and reflections. Data analysis followed the three-level coding process (Corbin & Strauss, 1990) using NVivo 14 software. There are three coding stages. In the initial coding stage, the collected data were subjected to open coding to identify the main concepts and themes (Timonen et al., 2018). Through axial coding, these concepts and themes were categorized and associated to form core categories (Charmaz & Thornberg, 2021). In the selective coding stage, these categories were further refined and integrated to form a theoretical model (Birks et al., 2019). Two researchers independently coded the data, discussed differences until consensus was reached, and maintained analytic memos throughout the process. Peer debriefing and member checking were used to strengthen the credibility and consistency of the analysis.

During open coding, meaningful expressions in the transcripts were labeled with initial concepts. For example, the sentence “Expert teachers have solid and systematic knowledge of the English language” (T16) was coded as “English Linguistic Knowledge”. In axial coding, similar concepts were grouped into broader categories, such as integrating “English Linguistic Knowledge” into the category “Linguistic Knowledge”. Through selective coding, these categories were further integrated into the five dimensions identified in this study. Table 1 presents several representative extracts to show how original interview statements were transformed into concepts, categories, and main categories. More detailed coding information is provided in Tables S2 and S3 in the Supplementary Materials to illustrate each dimension.

### 3.4. Theoretical Saturation Test

In accordance with grounded theory (Corbin & Strauss, 1990), theoretical saturation was tested to verify the adequacy of the model. A subset of interview transcripts was deliberately reserved for this purpose. These transcripts were then analyzed again to see whether any additional categories appeared. It was found that no new concepts emerged, and all findings were consistent with the existing framework. Through this process, the grounded theory approach provided both conceptual coherence and empirical richness in revealing the dimensions of college English teachers’ competence in the AI era.

## 4. Results

This section presents the findings generated from the grounded theory analysis, which identified five interrelated dimensions that constitute college English teachers’ adaptive expertise in the context of AI-empowered educational transformation. These dimensions are knowledge expertise, competence expertise, vision expertise, emotional expertise, and development expertise, which emerged from repeated coding and analysis of 40 interview transcripts.

### 4.1. Knowledge Expertise

Knowledge expertise forms the cognitive foundation of adaptive expertise. Participants consistently emphasized the importance of systematic disciplinary knowledge as well as interdisciplinary understanding. As one participant noted, “Expert teachers have systematic linguistic knowledge of English” (T16), highlighting the value of deep disciplinary mastery. Others emphasized the need to “possess a wide range of liberal arts knowledge” (T40), reflecting the interdisciplinary nature of modern English education.

Consistent with Shulman’s model of teacher knowledge (Shulman, 1987), participants viewed educational psychology and pedagogy as indispensable complements to subject matter expertise. They emphasized that effective English teaching requires integrating language knowledge with insights from educational psychology, curriculum design, and assessment. Such integration allows teachers to bridge theory and practice, enhancing their capacity to respond to diverse classroom contexts. In the context of AI-enhanced teaching environments, this flexible application of knowledge supports innovation and adaptation, serving as the foundation for other dimensions of adaptive expertise.

### 4.2. Competence Expertise

Competence expertise represents teachers’ practical ability to transform knowledge into effective pedagogical actions. Participants described competence in terms of teaching design, classroom implementation, evaluation, communication, and adaptability. For instance, one teacher mentioned, “I can set teaching objectives accurately and adjust the plan flexibly in class” (T16), while another emphasized “responding quickly to emergencies during lessons” (T24). These excerpts reveal that competence involves not only technical skills but also situational responsiveness and professional judgment.

Several teachers also discussed the need for technical and research competence, recognizing that AI-based learning platforms, automated assessment tools, and digital content creation have become integrated into classroom activities. As one participant observed, “We must learn to integrate new technologies into our teaching” (T9). Thus, competence expertise in the AI era extends beyond traditional pedagogy to include digital fluency and reflective application of technology for effective learning outcomes.

### 4.3. Vision Expertise

Vision expertise captures teachers’ capacity to perceive, interpret, and anticipate broader educational, cultural, and technological trends. Many participants emphasized the importance of a global outlook and cross-cultural awareness. One teacher remarked, “University English teachers can share and analyze international news in class” (T24), while another expressed the value of “cultural sensitivity and international vision” (T8). These reflections illustrate teachers’ awareness of globalization and their efforts to cultivate students’ intercultural competence.

Participants also demonstrated future-oriented thinking, frequently referencing “attention to the latest educational technologies and concepts” (T40). This forward-looking mindset enables teachers to align their instruction with societal and technological developments. Vision expertise, therefore, not only broadens teachers’ professional horizons but also empowers them to guide students to become globally competent individuals.

### 4.4. Emotional Expertise

Emotional expertise refers to teachers’ emotions and motivational resources that sustain engagement and resilience. Several teachers linked their sense of duty and satisfaction to their professional identity: “Education is also a responsibility for students’ growth” (T24), and “When facing setbacks, I usually remain optimistic” (T1). Such expressions reveal that emotional strength—grounded in a sense of responsibility, happiness, and self-efficacy—underpins teachers’ capacity to adapt and persevere in the face of challenges.

Emotional expertise also influences classroom climate and teacher–student relationships. Participants described how a positive emotional state enables them to inspire students and navigate the emotional complexities of teaching. In the fast-changing AI-empowered context, such emotional adaptability becomes crucial for sustaining professional commitment and well-being.

### 4.5. Development Expertise

Development expertise embodies teachers’ continuous pursuit of growth through reflection, learning, and innovation. Teachers frequently mentioned “lifelong learning,” “self-reflection,” and “pursuing innovation” as teachers’ core literacy. One participant stated, “I often reflect on my teaching after class to find better ways for improvement” (T1). Another added, “We should always learn from new teaching methods and adapt to new technologies” (T2). These statements demonstrate a proactive stance toward professional renewal and adaptation.

This dimension aligns with the notion of sustainable professional development, emphasizing that adaptive expertise is not a static trait but a dynamic process. Teachers who constantly learn, experiment, and reflect are better equipped to engage with educational reforms and technological change. Development expertise thus drives the ongoing evolution of teachers’ professional competence.

### 4.6. Theoretical Model of Adaptive Expertise

**Figure 1:**
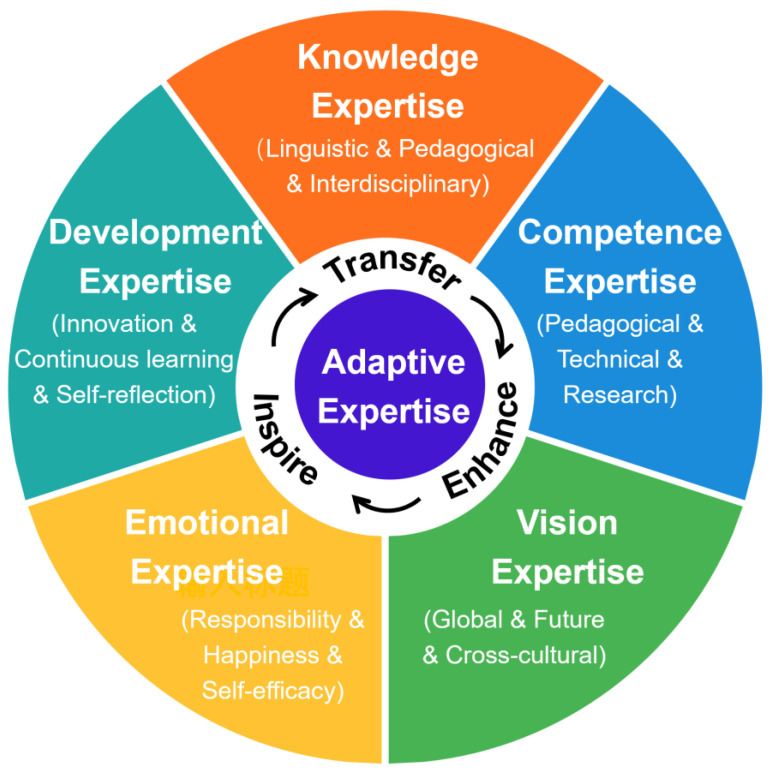
Theoretical model of adaptive expertise among college English teachers.

The five dimensions identified in this study were further integrated into a theoretical model that explains how college English teachers develop and enact adaptive expertise in AI-enhanced educational environments. As illustrated in Figure 1, the dimensions do not function independently but operate as an interconnected and dynamic system that supports teachers’ ongoing adaptation to technology-mediated teaching contexts.

Knowledge expertise provides the conceptual foundation for teaching. It helps teachers understand how AI-related innovations reshape instructional requirements and decide how intelligent tools can be incorporated to support language learning. When applied in real teaching situations, knowledge naturally extends into competence expertise, where teachers design, implement, and refine technology-supported instructional practices. These practical experiences, in turn, deepen and reorganize teachers’ knowledge, forming a reciprocal relationship between knowing and doing.

Competence expertise transforms that knowledge into effective pedagogical practice. Knowledge attains its full value only when it is effectively transferred into professional competence. In the AI era, technological and pedagogical abilities enable teachers to apply their knowledge in teaching and research, thereby enhancing their adaptive expertise. In this sense, competence expertise not only transfers knowledge into action but also shapes how knowledge evolves, influences teachers’ future-oriented vision, and deepens their commitment to continuous development.

Vision expertise provides direction for teachers’ professional development. It broadens teachers’ global and future-oriented perspectives. By staying attentive to global trends and developments in AI-assisted communication, teachers are able to anticipate new requirements in English education. This forward-looking orientation influences how knowledge is updated and how pedagogical choices are made.

Emotional expertise sustains teachers’ motivation, engagement, and resilience. It maintains the psychological conditions under which adaptation can occur. Teachers’ sense of responsibility, self-efficacy, and emotional stability help them navigate uncertainty, manage the pressures brought by technological change, and remain committed to their work. Consequently, emotional expertise functions as an inspiring force: it affects how confidently teachers apply knowledge alongside intelligent tools, how flexibly they respond to AI-related challenges, and how consistently they pursue development in AI-mediated teaching contexts.

Development expertise links the system together by transforming individual adjustments into continuous improvement in AI-enhanced teaching contexts. It enables teachers to turn short-term adaptations, such as learning to use new AI tools and adjusting to data-informed instructional demands, into sustained professional development. By drawing on knowledge, competence, vision, and emotional resources, teachers integrate insights from the other dimensions through reflection, learning, and innovation, gradually building a more resilient professional identity to withstand rapid AI-driven shifts.

In sum, the five dimensions function as a fluid and mutually reinforcing system driven by the adaptive processes of transfer, enhance, and inspire. Knowledge enables action; competence refines and deepens understanding; vision guides their direction; emotional resources support sustained engagement; and development expertise carries the process forward over time. Together, they form a coherent and dynamic mechanism through which teachers continually reconstruct their professional competence in AI-enhanced educational environments.

## 5. Discussion

China’s college English education is undergoing a profound transformation due to curriculum reform and the accelerating integration of artificial intelligence (AI) technologies. While expertise has long been conceptualized as a continuous pursuit of excellence (Tsui, 2009), how teachers renew such excellence under dynamic and technologically mediated conditions remains underexplored. This gap has been confirmed by recent studies showing that research on AI in education has focused predominantly on technological applications in teaching, while comparatively limited attention has been paid to how teachers reconstruct professional competence in response to AI integration (Tan et al., 2025). The present study addresses this imbalance by conceptualizing adaptive expertise as a professional capacity that emerges as teachers rethink their roles, knowledge, and professional growth in response to AI-supported teaching environments. Previous research has discussed adaptive expertise largely in general terms (Anthony et al., 2015), but few have examined its internal structure within the specific context of English teaching in China. Using the grounded theory approach, this study derived theoretical understanding inductively from empirical data rather than pre-existing frameworks (Charmaz & Thornberg, 2021). Through open, axial, and selective coding, a conceptual model emerged that views adaptive expertise as a dynamic, evolving capacity rather than a fixed set of skills.

Consistent with earlier studies emphasizing knowledge and competence (Anthony et al., 2015; Gube & Lajoie, 2020; Leikin & Ovodenko, 2024; Moonthiya & Stevenson, 2024; Tan et al., 2025; Tay et al., 2025; Wang et al., 2025; Yang et al., 2024; Yue et al., 2024), this study confirmed that knowledge and competence are the practical core of teachers’ expertise. Unlike previous studies, this study focuses on the specific components of adaptive expertise for college English teachers and extends previous work by identifying emotional, visionary, and developmental dimensions as equally essential to adaptation in the AI era. Emotions, both positive and negative, are integral to the teaching experience, as they influence how teachers prepare and deliver instruction, navigate the curriculum, and interact with their students (Richards, 2022). Vision expertise involves global and future awareness, as well as intercultural sensitivity. There is a growing incorporation of research-based teacher education programs intended to help future teachers acquire an investigative, multifaceted approach to teaching, including the development of teachers’ professional vision (Finefter-Rosenbluh & Power, 2023). English teachers feel they need training and development within the broader academy (Fitzpatrick et al., 2022). Development expertise provides direction for the sustainable development of teachers. Development expertise not only reflects the continuity of teachers’ professional development but also lays the foundation for their long-term competitiveness in the education industry. The five constituent elements provide references and insights for the specialized dimensions of adaptive expertise for other disciplines.

In addition, the findings of this study are consistent with research on language teacher cognition, which emphasizes that teachers’ beliefs and knowledge structures shape their pedagogical reasoning and classroom decisions (Borg, 2003). The dimensions demonstrate how teachers draw upon a complex cognitive system to interpret instructional demands, negotiate institutional expectations, and respond to technological shifts. At the same time, the model resonates with studies on technology integration in language education. Mishra and Koehler (2006) highlight tech–pedagogical judgment which matches competence expertise. Warschauer (2011) emphasizes digital literacy, echoing knowledge and vision expertise. Lai (2015) stresses continual tech-based learning that supports the development of expertise. These findings indicate that teacher adaptability in the AI era is a cognitive–emotional process that shapes how teachers interpret technological change and refine their professional practice.

### 5.1. The Adaptive and Dynamic Nature of the Five Dimensions

Each dimension embodies adaptability in a unique and interconnected way. Knowledge expertise is adaptive in its evolutionary nature as teachers integrate disciplinary, pedagogical, and technological knowledge to meet evolving learning demands. In AI-mediated environments, for instance, teachers reinterpret language knowledge to integrate digital tools and multi-modal literacy. Competence expertise reflects situational adaptability, as teachers redesign instruction, assessment, and communication strategies to fit new technologies, hybrid classrooms, and diverse learners. This aligns with Hatano and Inagaki’s (1986) notion that adaptive experts flexibly apply knowledge to AI-enhanced settings. Vision expertise embodies anticipatory adaptability. Teachers cultivate global and future-oriented awareness to anticipate technological trends, engage intercultural perspectives, and prepare students for AI-driven communication. Emotional expertise represents psychological adaptability and is often activated when teachers confront AI-mediated change. Emerging evidence from both language education and workplace studies suggests that AI-related contexts reshape how professionals interpret feedback, regulate emotions, and adjust their professional learning, with emotional experiences dynamically interacting with professional development to foster adaptive expertise rather than merely accompanying technological adoption (Liao et al., 2026; Liu & Chang, 2024). These findings help explain why emotional expertise emerged in the present model as a necessary condition for adaptive expertise rather than as a secondary personal attribute. Self-efficacy and a sense of responsibility and happiness enable teachers to manage uncertainty, technological anxiety, and professional stress, sustaining motivation in an era of constant change. Development expertise is the sustainability engine of adaptation. Teachers can keep developing and remain invincible through lifelong learning, innovation, and reflective practice in the AI era.

### 5.2. Integrating the Five Dimensions into an AI-Era Model

The final theoretical model (Figure 1) synthesizes these five dimensions into an integrated system where each domain informs and strengthens the others.

Knowledge provides the cognitive foundation for informed decision-making. Competence transforms theoretical understanding into pedagogical action. Vision situates teaching within global, technological, and intercultural contexts. Emotion maintains motivation and ethical engagement. Development ensures sustainability and innovation across the professional lifespan.

The interplay among these domains forms an adaptive cycle: knowledge informs competence; competence, when enacted reflectively, enhances knowledge; vision guides both knowledge and competence; emotional expertise sustains the cycle; and developmental expertise ensures ongoing renewal. These dimensions transfer, enhance, and inspire one another. This cyclical and interactive structure captures the core of adaptability—continuous reconstruction, reflection, and renewal in the face of change.

### 5.3. Theoretical Significance and Practical Implications

This study contributes to the literature on adaptive expertise by providing a discipline-specific and empirically grounded model of adaptive expertise among college English teachers in the AI era. Unlike prior studies that conceptualize adaptive expertise mainly in general terms, this research clarifies its internal structure within the context of language education in Chinese higher education. By identifying five interrelated dimensions, the study extends existing frameworks on adaptive expertise (Bransford et al., 2000; Carbonell et al., 2014) and illustrates how teacher adaptability unfolds as a dynamic and developmental process under conditions of technological transformation. The findings underscore that adaptive expertise is a multidimensional system shaped by a solid knowledge base, context-sensitive instructional competence, emotional regulation, future-oriented vision, and sustained professional growth, echoing prior research that conceptualizes teacher expertise as dynamic and context-sensitive rather than static (Tsui, 2009). The findings of this study offer meaningful implications for teacher education and professional development in the AI era. Universities and teacher training institutions may use the proposed model as a reference framework to design professional development programs that go beyond technical skill training and long-term career development. In particular, targeted support for AI-enhanced instructional competence, opportunities for cultivating global and future-oriented professional vision, and institutional mechanisms that encourage lifelong learning may help college English teachers sustain adaptability in rapidly changing educational environments. More broadly, the model may serve as a conceptual reference for examining adaptive expertise in other disciplines confronted with comparable technological and curricular transformations.

## 6. Conclusions

This study employed grounded theory to explore the adaptive expertise of Chinese college English teachers in the context of the AI-driven transformation of higher education. Based on interviews with 40 teachers, it constructed a dynamic theoretical model that reveals the internal structure and formation mechanism of teachers’ adaptive expertise in response to the dual challenges of curriculum reform and technological transformation. The model comprises five interrelated dimensions: knowledge expertise (the foundation), competence expertise (the core), vision expertise (the expansion), emotional expertise (the driving force), and development expertise (the direction). They constitute a comprehensive framework that captures how teachers sustain effective teaching and professional growth in the age of artificial intelligence.

Theoretically, this model extends existing studies on adaptive expertise (Anthony et al., 2015; Gube & Lajoie, 2020; Leikin & Ovodenko, 2024; Moonthiya & Stevenson, 2024; Tan et al., 2025; Tay et al., 2025; Wang et al., 2025; Yang et al., 2024; Yue et al., 2024) by clarifying how the five dimensions operate interactively in the digital and interdisciplinary context. It shows that teachers’ professional adaptability is not static but dynamic—continuously reconstructed through reflection, technological integration, and emotional regulation.

Practically, the study provides actionable implications for teacher development in the AI era. Universities should design training systems that foster teachers’ ability to integrate intelligent technologies, maintain emotional balance, and continuously reflect on practice. The model also offers a reference framework for developing AI-resilient pedagogical expertise across disciplines.

Nevertheless, this study has its limitations. It focuses on the qualitative identification of the components and relationships within adaptive expertise but does not empirically test their effects on teaching performance. Future research should quantitatively validate this model, explore how adaptive expertise evolves over time, and conduct comparative studies across different cultural and institutional contexts. Such efforts will further clarify how teachers can retain human-centered professionalism while effectively co-evolving with intelligent technologies in higher education.

## Figures and Tables

**Table 1:** Representative Examples of Open, Axial, and Selective Coding.

Original Interview Statement	Concept	Category	Main Category
“Expert teachers have solid and systematic knowledge of the English language.” (T16)	English Linguistic Knowledge	Linguistic Knowledge	Knowledge Expertise
“Learn new skills such as the ability to integrate information technology with teaching.” (T39)	Information Technology Application	Technical Competence	Competence Expertise
“Have a certain degree of cultural sensitivity and international vision.” (T40)	Cultural Sensitivity	Cross-cultural Vision	Vision Expertise
“I believe that as long as I keep working hard, I can overcome difficulties and improve my teaching level.” (T24)	Self-motivation	Self-efficacy	Emotional Expertise
“Establish the concept of lifelong learning and continuously improve professional quality.” (T2)	Lifelong Learning	Continuous Learning and Adaptation	Development Expertise

## Data Availability

All data are available upon request.

## Supplementary Materials

The following supporting information can be downloaded at https://www.mdpi.com/article/10.3390/bs16040476/s1.

## References

[B1-behavsci-16-00476] ACTFL (2012). ACTFL proficiency guidelines 2012.

[B2-behavsci-16-00476] Anthony G., Hunter J., Hunter R. (2015). Prospective teachers development of adaptive expertise. Teaching and Teacher Education.

[B3-behavsci-16-00476] Birks M., Hoare K., Mills J. (2019). Grounded theory: The Faqs. International Journal of Qualitative Methods.

[B4-behavsci-16-00476] Borg S. (2003). Teacher cognition in language teaching: A review of research on what language teachers think, know, believe, and do. Language Teaching.

[B5-behavsci-16-00476] Bransford J. D., Brown A. L., Cooking R. R. (2000). How people learn: Brain, mind, experience, and school.

[B6-behavsci-16-00476] Byram M. (1997). Teaching and assessing intercultural communicative competence.

[B7-behavsci-16-00476] Carbonell K. B., Stalmeijer R. E., Konings K. D., Seger M., van Merrienboer J. J. G. (2014). How experts deal with novel situations: A review of adaptive expertise. Educational Research Review.

[B8-behavsci-16-00476] Charmaz K., Thornberg R. (2021). The pursuit of quality in grounded theory. Qualitative Research in Psychology.

[B9-behavsci-16-00476] Corbin J., Strauss A. (1990). Grounded theory research—Procedures, canons and evaluative criteria. Zeitschrift Fur Soziologie.

[B10-behavsci-16-00476] Cresswell J. (2013). Qualitative inquiry & research design: Choosing among five approaches.

[B11-behavsci-16-00476] Farrell T. (2018). Reflective practice in language teaching: Research-based principles and practices.

[B12-behavsci-16-00476] Finefter-Rosenbluh I., Power K. (2023). Exploring preservice teachers’ professional vision: Modes of isolation, ethical noticing, and anticipation in research communities of practice. Teaching and Teacher Education.

[B13-behavsci-16-00476] Fitzpatrick D., Costley T., Tavakoli P. (2022). Exploring Eap teachers? Expertise: Reflections on practice, pedagogy and professional development. Journal of English for Academic Purposes.

[B14-behavsci-16-00476] Gube M., Lajoie S. (2020). Adaptive expertise and creative thinking: A synthetic review and implications for practice. Thinking Skills and Creativity.

[B15-behavsci-16-00476] Hatano G., Inagaki K., Stevenson H., Azuma H., Hakuta K. (1986). Two courses of expertise. Child development and education in Japan.

[B16-behavsci-16-00476] Holmes W., Bialik M., Fadel C. (2019). Artificial intelligence in education promises and implications for teaching and learning.

[B17-behavsci-16-00476] Lai C. (2015). Modeling teachers’ influence on learners’ self-directed use of technology for language learning outside the classroom. Computers & Education.

[B18-behavsci-16-00476] Lee I., Yuan R. E. (2021). Understanding L2 writing teacher expertise. Journal of Second Language Writing.

[B19-behavsci-16-00476] Leikin R., Ovodenko R. (2024). Math-light problem posing by three experts with different fields of expertise: Why? what? and how?. The Journal of Mathematical Behavior.

[B20-behavsci-16-00476] Liao G., Ren X., Zheng X., Zhang Y. (2026). Shame or anger? The impact of negative performance feedback sources (Ai versus leader) on employees’ job crafting. Behavioral Sciences.

[B21-behavsci-16-00476] Lin X., Schwartz D. L., Hatano G. (2005). Toward teachers’ adaptive metacognition. Educational Psychologist.

[B22-behavsci-16-00476] Liu Y., Chang P. (2024). Exploring EFL teachers’ emotional experiences and adaptive expertise in the context of Ai advancements: A positive psychology perspective. System.

[B23-behavsci-16-00476] Mercier E. M., Higgins S. E. (2013). Collaborative learning with multi-touch technology: Developing adaptive expertise. Learning and Instruction.

[B24-behavsci-16-00476] Ministry of Education (2020). Guidelines for college english teaching.

[B25-behavsci-16-00476] Mishra P., Koehler M. J. (2006). Technological pedagogical content knowledge: A framework for teacher knowledge. Teachers College Record.

[B26-behavsci-16-00476] Moonthiya I., Stevenson M. (2024). Identities of non-English-dominant teachers in transnational language teacher education: A systematic review. Teaching and Teacher Education.

[B27-behavsci-16-00476] Priestley M. R., Biesta G., Robinson S. (2015). Teacher agency: An ecological approach.

[B28-behavsci-16-00476] Richards J. C. (2022). Exploring emotions in language teaching. Relc Journal.

[B29-behavsci-16-00476] Shulman L. S. (1987). Knowledge and teaching—Foundations of the new reform. Harvard Educational Review.

[B30-behavsci-16-00476] Tan X., Cheng G., Ling M. H. (2025). Artificial intelligence in teaching and teacher professional development: A systematic review. Computers and Education: Artificial Intelligence.

[B31-behavsci-16-00476] Tay P. L. L., Seah L. H., Chia T. T. (2025). Assessing science teachers’ adaptive expertise in teaching disciplinary literacy using the Aedli framework. Teaching and Teacher Education.

[B32-behavsci-16-00476] TESOL (2018). Standards for Esl/Efl teachers of adults.

[B33-behavsci-16-00476] Timonen V., Foley G., Conlon C. (2018). Challenges when using grounded theory: A pragmatic introduction to doing gt research. International Journal of Qualitative Methods.

[B34-behavsci-16-00476] Tsui A. B. M. (2009). Distinctive qualities of expert teachers. Teachers and Teaching.

[B35-behavsci-16-00476] Wang X., Husu J., Toom A. (2025). What makes a good mentor of in-service teacher education?—A systematic review of mentoring competence from a transformative learning perspective. Teaching and Teacher Education.

[B36-behavsci-16-00476] Warschauer M. (2011). Learning in the cloud: How (and why) to transform schools with digital media.

[B37-behavsci-16-00476] Yang Y., Tseng C. C., Lai S. (2024). Enhancing teachers’ self-efficacy beliefs in ai-based technology integration into English speaking teaching through a professional development program. Teaching and Teacher Education.

[B38-behavsci-16-00476] Yue W., Yan L., Yang Y. (2024). How exactly do teachers’ identities develop in the study travel?—A grounded theory study from China. Teaching and Teacher Education.

[B39-behavsci-16-00476] Zawacki-Richter O., Mar I N V. I., Bond M., Gouverneur F. (2019). Systematic review of research on artificial intelligence applications in higher education—Where are the educators?. International Journal of Educational Technology in Higher Education.

