# Supplementary material for: Adaptive Expertise of College English Teachers in the Era of Artificial Intelligence: A Grounded Theory Approach
Source: Behav Sci (Basel). 2026 Mar 24;16(4):476. doi: 10.3390/bs16040476 (PMC13113875; doi:10.3390/bs16040476)
Supplement: Supplementary file 1 [file behavsci-16-00476-s001.zip › behavsci-4015978-supplementary.pdf]

Supplementary Materials

S1. Coding

Table S1. Basic Information of Respondents

Code	Gender	Age	Education	Title	Years of Teaching
T1	Male	30	Master's	Assistant Professor	2
T2	Female	30	Master's	Lecturer	4
T3	Female	35	Master's	Lecturer	4
T4	Female	29	Master's	Lecturer	5
T5	Female	31	Master's	Lecturer	6
T6	Female	34	Master's	Lecturer	7
T7	Male	33	Master's	Lecturer	7
T8	Female	35	Master's	Lecturer	8
T9	Female	38	Master's	Lecturer	11
T10	Female	38	Master's	Lecturer	13
T11	Female	41	Master's	Lecturer	13
T12	Male	48	Master's	Lecturer	14
T13	Female	42	Master's	Associate Professor	14
T14	Female	41	Master's	Lecturer	15
T15	Female	41	Master's	Lecturer	15
T16	Female	41	Master's	Lecturer	15
T17	Male	43	Master's	Lecturer	15
T18	Male	42	Master's	Lecturer	15
T19	Male	42	Master's	Lecturer	15
T20	Male	42	Doctorate	Lecturer	15
T21	Male	50	Master's	Associate Professor	16

T22	Female	41	Master's	Lecturer	16
T23	Female	44	Master's	Lecturer	17
T24	Female	42	Master's	Lecturer	17
T25	Female	42	Master's	Associate Professor	17
T26	Female	47	Master's	Associate Professor	17
T27	Female	47	Master's	Lecturer	17
T28	Female	42	Doctorate	Professor	17
T29	Female	42	Master's	Associate Professor	18
T30	Male	41	Master's	Associate Professor	19
T31	Female	40	Master's	Associate Professor	19
T32	Female	45	Doctorate	Associate Professor	19
T33	Female	46	Master's	Associate Professor	20
T34	Female	43	Master's	Lecturer	20
T35	Female	49	Master's	Associate Professor	22
T36	Female	47	Master's	Associate Professor	23
T37	Female	47	Master's	Associate Professor	23
T38	Female	50	Doctorate	Professor	25
T39	Female	61	Master's	Associate Professor	39
T40	Female	60	Master's	Professor	40

Table S2. Examples of Open Coding.

Original Interview Statement	Concept	Category
T16：Expert teachers have solid and systematic knowledge of English language.	English Linguistic Knowledge	Linguistic Knowledge
T40：Able to teach students the essence of the English language and have a profound	English Literary Knowledge	

knowledge of English literature.		
T40: Expert teachers should have English translation knowledge, teach students translation theories, and guide them in translation practice.	English Translation Knowledge	
T40： Possess educational psychology knowledge, and can use educational psychology theories to guide teaching, flexibly adjusting teaching methods according to students' different needs.	Educational Psychology Knowledge	Pedagogical Knowledge
T24: Have a rich knowledge of educational psychology theories and practical knowledge, and be able to better understand the psychological problems existing in students' learning process and adjust teaching strategies in time.	Application of Educational Psychology Knowledge	
T40: Possess a wide range of liberal arts knowledge.	Liberal Arts Knowledge	Interdisciplinary Knowledge
T40: University English teachers who teach mathematics majors can teach students the corresponding English mathematical terms and have a profound knowledge of science.	Science Knowledge	
T40: Teachers who teach students majoring in mechanical engineering know mechanical English terms and have knowledge of engineering.	Engineering Knowledge	
T16: Able to set teaching objectives accurately, not only focusing on language knowledge and skills, but also involving the cultivation of comprehensive qualities such as cultural awareness and thinking quality.	Teaching Objective Setting	Teaching Design Competence

T16: Expert teachers have proficient teaching skills and can design reasonable teaching plans according to different teaching content and students' characteristics.	Organization of Teaching Content	
T16: They are good at organizing classroom teaching and have strong classroom management skills.	Classroom Management	Teaching Implementation Competence
T16: Expert teachers can keenly perceive differences in students' learning styles, abilities, and progress, and then adopt diversified teaching strategies to meet the needs of different students.	Personalized Teaching	
T40: In the classroom, they can better guide students to participate in discussions and stimulate their interest.	Classroom Interaction	
T16: Flexible use of various teaching methods, such as communicative teaching method and task-based teaching method.	Teaching Strategies	
T1: They have a more comprehensive grasp of English subject knowledge and can integrate language knowledge with cultural knowledge in teaching.	Knowledge Integration Teaching	
T35: In terms of post-class teaching evaluation, novice teachers may omit students' feedback. Expert teachers, on the other hand, will have more thoughtful considerations and practices in these aspects.	Student Performance Evaluation	Teaching Assessment Competence
T40: After class, they will also give students timely feedback to help them improve their learning.	Teaching Effect Feedback	

T8: Be good at communicating with students, able to stimulate their autonomy and interest in learning, and achieve mutual trust and understanding with students.	Communication Skills	Social competence
T24: Expert teachers often have stronger intuitive judgment abilities and can quickly respond to emergencies in the classroom.	Emergency Handling	Adaptability and Coordination Competence
T40: Conflict resolution ability is reflected in teachers' ability to effectively deal with disputes among students or differences of opinion in the classroom, creating a harmonious learning atmosphere.	Conflict Resolution	
T16: Able to adjust the teaching progress in time according to students' classroom reactions, such as slowing down and adding case explanations when finding students have difficulty understanding a certain knowledge point.	Classroom Adaptation and Adjustment	
T40: Adapt to the constantly changing external environment through continuous learning, participating in professional development activities, and actively adapting to new technologies and teaching models.	Environmental Adaptation	
T25: Establish good cooperative relationships with colleagues, students, and educational managers to jointly face the challenges of educational reform and innovation. Through teamwork, teachers can share resources, exchange experiences, and improve teaching quality together.	Teamwork	

T40: University English teachers need to have a spirit of teamwork.	Teamwork Spirit	
T24: Able to apply for research projects and publish related academic papers.	Project Application Competence	Research Competence
T40: Expert teachers not only have rich experience and ability in applying for projects, but also are good at publishing academic papers.	Paper Publishing Competence	
T39: Learn new skills such as the ability to integrate information technology with teaching.	Information Technology Application	Technical Competence
T6: Able to use modern educational technology and multimedia resources to improve teaching effectiveness.	Digital Resource Utilization	
T8: Able to skillfully use various learning platforms to enhance teaching effectiveness.	Online Teaching Platforms	
T24: University English teachers can share and analyze International hot news with students to cultivate students' international vision.	International Hot News	Global Vision
T40: Keep up with international academic trends, participate in academic exchanges and have a global vision.	International Academic Trends	
T25: Expert teachers generally have rich teaching experience and will also carry out teaching and research activities in combination with their own academic interests and professional abilities, paying attention to the frontier development of the subject and educational teaching	Frontier Development of the Subject	Future Vision

methods.		
T40: Pay attention to the latest educational technology and teaching concepts.	Advanced Teaching Concepts	
T23: University English teachers should maintain a keen perception of the education industry and technology, continuously learn and update their knowledge and skills.	Perception of Future Technology	
T8: Have a certain degree of cultural sensitivity and international vision.	Cultural Sensitivity	Cross-cultural Vision
T8: Help students better understand cultural differences and cultivate their cross-cultural communication abilities.	Multicultural Understanding	
T6: Have cross-cultural communication abilities.	Cross-cultural Communication	
T24: Education is not only the transmission of knowledge, but also a responsibility centered on students' growth.	Sense of Educational Mission	Sense of Responsibility
T14: Outstanding teachers not only focus on academic education, but also pay more attention to students' moral cultivation.	Educational Commitment	
T24: Have a clear goal for one's own career development, and be able to better plan one's time to achieve the career dreams.	Career Pursuit	
T24: It is not only a means of livelihood, but also a responsibility of educating people.	Sense of Educational Responsibility	
T1: When facing setbacks and pressures, I usually remain optimistic.	Positive Attitude	Sense of Happiness

T38: Teachers should have passion and sense of responsibility for the education cause.	Teaching Enthusiasm	
T16: If facing setbacks that can be endured, I will devote myself to completing it.	Teaching Confidence	Self-efficacy
T24: I believe that as long as I keep working hard, I can overcome difficulties and improve my teaching level.	Self-motivation	
T24: Able to continuously improve teaching quality through persistent efforts, and gain a high sense of achievement from students' learning outcomes.	Pursuit of Achievement	
T7: University English teachers need to continuously learn, integrate technology, engage in cross-cultural teaching, innovate in teaching, and cooperate in teams to adapt to changes in the educational environment.	Teaching Innovation	Pursuit of Innovation
T1: Participate more in academic seminars and research projects, and inject new ideas into one's academic research by learning new research methods and cutting-edge achievements, achieving academic innovation.	Academic Innovation	
T1: Learn more modern intelligent teaching tools, such as using artificial intelligence systems to provide personalized learning feedback, optimize students' learning experience and efficiency, and improve teaching effectiveness, achieving technical innovation.	Technical Innovation	
T1: University English teachers should have an	Thinking Innovation	

innovative spirit, be good at cultivating students' critical and creative thinking through open discussions and guiding questions, encourage students to analyze problems from multiple perspectives, and improve problem-solving abilities by applying practical cases in the classroom, achieving thinking innovation.		
T2: Establish the concept of lifelong learning, continuously improve one's professional quality and teaching skills, and actively participate in various professional development activities.	Lifelong Learning	Continuous Learning
T9: There should be continuous self-development, continuous learning and updating of teaching concepts and methods, participation in professional development activities to adapt to the constant changes in education.	Professional Growth	
T2: Actively participate in professional training, academic exchanges, and educational research to continuously enhance one's professional competence and teaching proficiency.	Training Participation	
T1: Expert teachers reflect on the effects of their teaching after class.	Teaching Reflection	Self-reflection
T30: Able to continuously evaluate one's own teaching practice.	Self-assessment	
T6: Possess critical thinking skills.	Critical thinking	

Table S3. Results of Axial Coding.

Main Category	Category	Description
Knowledge Expertise	Linguistic Knowledge	The systematic knowledge of teachers in areas such as English linguistics, teaching methodology theory, and cultural studies, which forms the theoretical basis of teaching activities.
	Pedagogical Knowledge	Knowledge involving areas such as educational psychology, educational philosophy, and educational assessment, providing teachers with theoretical support and methodological guidance in educational practice.
	Interdisciplinary Knowledge	The integration of knowledge from multiple disciplines to cultivate teachers' interdisciplinary vision and comprehensive teaching abilities.
Competence Expertise	Pedagogical Competence	Teachers’ ability to evaluate student performance and feedback on teaching effectiveness, with the key being the ability to adjust teaching strategies based on assessment results.
	Technical Competence	Teachers’ ability to use information technology, digital resources, and online teaching platforms, demonstrating their capabilities in technology integration and teaching modernization.
	Research Competence	Teachers’ comprehensive skills in research innovation, research design, literature review, data analysis, and academic writing, reflecting their ability to apply theoretical knowledge to

		practical research.
Vision Expertise	Global Vision	Teachers’ understanding and attention to international educational trends and global educational issues, reflecting their international vision and global awareness.
	Future Vision	Teachers’ perception and anticipation of the frontier of the subject, advanced teaching concepts, and future technologies, demonstrating their forward-looking and innovative consciousness.
	Cross-cultural Vision	Teachers’ abilities in cultural sensitivity, multicultural understanding, and cross-cultural communication, which are crucial for cultivating students with an international vision.
Emotional Expertise	Sense of Responsibility	Teachers’ deep affection for and noble pursuit of the education cause, including a profound understanding of educational values, commitment to students’ holistic development, and innovation and leadership in educational practice.
	Sense of Happiness	Teachers’ intrinsic motivation for teaching, pursuit of career development, passion for teaching work, and willingness to pursue personal achievement.
	Self-efficacy	Teachers’ confidence in their teaching abilities, capacity to cope with challenges, self-motivation to achieve teaching goals, and ability to maintain a positive attitude in adversity.
Development Expertise	Pursuit of Innovation	Reflects teachers' willingness and ability to constantly seek new ideas, methods, and

		technologies in educational practice, emphasizing the spirit of innovation in teaching content, methods, and technology, as well as adaptability and flexibility.
	Continuous Learning and Adaptation	Teachers' willingness and ability in lifelong learning, professional growth, skill expansion, and training participation, which is crucial for teachers to adapt to educational changes and continuous development.
	Self-reflection	Teachers' abilities in teaching reflection, self-assessment, and critical thinking, which play an important role in promoting teachers' professional growth.

S2. Interview Outline

Part I. Background Information

1. Institution type

2. Gender

3. Age range

4. Highest academic degree

5. Professional title

6. Years of teaching experience in higher education

Part II. Interview Questions

1. In your experience, how do college English teachers differ in terms of knowledge and professional competence across different stages of their careers?

2. Beyond knowledge and skills, what other differences do you observe among college

English teachers with varying levels of experience?

3. Can you describe how college English teachers may differ in their teaching practices before, during, and after class?

4. In your view, what characteristics define an excellent college English teacher in the current educational context? Please describe your understanding in detail.

5. How do you think college English teachers adapt to changes in their teaching environment, such as curriculum reform or the introduction of new technologies?

6. Do you have a personal plan for your professional development? How do you understand the role of career planning in teachers' long-term growth?

7. How would you describe your emotional engagement with teaching? What factors influence your motivation to continue developing as a teacher?

8. When you encounter pressure or setbacks in your work, how do you usually respond? What helps you cope with these challenges?

9. How do family, colleagues, institutional policies, or leadership support influence your teaching and professional development? Are there areas you think could be improved?

10. Can you share experiences in your teaching career that have brought you positive or negative emotional experiences? How have these experiences influenced your teaching practices?

11. Is there anything else you would like to share that we have not covered but that you consider important to your experiences as a college English teacher?
